# An efficient preparation of labelling precursor of [^11^C]L-deprenyl-D_2_ and automated radiosynthesis

**DOI:** 10.1186/s41181-017-0029-5

**Published:** 2017-07-24

**Authors:** Kevin Zirbesegger, Pablo Buccino, Ingrid Kreimerman, Henry Engler, Williams Porcal, Eduardo Savio

**Affiliations:** 1 Centro Uruguayo de Imagenología Molecular (CUDIM), Av. Dr. Américo Ricaldoni 2010, 11600 Montevideo, Uruguay; 2 Departamento de Química Orgánica, Facultad de Química, Universidad de la República, Montevideo, Uruguay; 3 Cátedra de Radioquímica, Facultad de Química, Universidad de la República, Montevideo, Uruguay

**Keywords:** L-nordeprenyl-D_2_, Organic precursor, [^11^C]L-deprenyl-D_2_, Automated synthesis, PET radiopharmaceutical

## Abstract

**Background:**

The synthesis of [^11^C]L-deprenyl-D_2_ for imaging of astrocytosis with positron emission tomography (PET) in neurodegenerative diseases has been previously reported. [^11^C]L-deprenyl-D_2_ radiosynthesis requires a precursor, L-nordeprenyl-D_2_, which has been previously synthesized from L-amphetamine as starting material with low overall yields. Here, we present an efficient synthesis of L-nordeprenyl-D_2_ organic precursor as free base and automated radiosynthesis of [^11^C]L-deprenyl-D_2_ for PET imaging of astrocytosis. The L-nordeprenyl-D_2_ precursor was synthesized from the easily commercial available and cheap reagent L-phenylalanine in five steps. Next, *N*-alkylation of L-nordeprenyl-D_2_ free base with [^11^C]MeOTf was optimized using the automated commercial platform GE TRACERlab® FX C Pro.

**Results:**

A simple and efficient synthesis of L-nordeprenyl-D_2_ precursor of [^11^C]L-deprenyl-D_2_ as free base has been developed in five synthetic steps with an overall yield of 33%. The precursor as free base has been stable for 9 months stored at low temperature (−20 °C). The labelled product was obtained with 44 ± 13% (*n* = 12) (end of synthesis, decay corrected) radiochemical yield from [^11^C]MeI after 35 min synthesis time. The radiochemical purity was over 99% in all cases and specific activity was (170 ± 116) GBq/μmol.

**Conclusions:**

A high-yield synthesis of [^11^C]L-deprenyl-D_2_ has been achieved with high purity and specific activity. L-nordeprenyl-D_2_ precursor as free amine was applicable for automated production in a commercial synthesis module for preclinical and clinical application.

## Introduction, background and literature review

Astrocytes become activated in response to many CNS pathologies such as stroke, trauma, growth of tumours or neurodegenerative diseases (Pekny and Nilsson 2005). Recent studies demonstrated that astrocytic MAO-B is increased in neurodegenerative diseases such as Parkinson and Alzheimer (Mallajosyula, et al. 2008; Gulyas et al. 2011). In this context, changes in concentrations of MAO-B have been proposed as an in vivo marker of neuroinflammation associated with Alzheimer’s disease (Rodriguez-Vieitez et al. 2015; Rodriguez-Vieitez et al. 2016). The distribution of the MAO-B enzyme in the brain of normal healthy volunteers and brains of patients with different pathologies has been studied with PET (Fowler et al. 2015). The PET tracer [^11^C]L-deprenyl-D_2_ binds selectively and irreversibly to the MAO-B (Fowler et al. 1987; Fowler et al. 2005).This compound acts as a suicide inhibitor of the MAO-B through a covalent linkage during normal catalytic stage, which involves cleavage of the C-D bond in the methylene carbon of the propargyl group (Fowler et al. 2015; Fowler et al. 2002). In the last years, this PET radiotracer has been applied to investigate astrocytosis in neurodegenerative diseases including Alzheimer’s disease, Creutzfeldt Jakob disease and Amiotrophic Lateral Sclerosis (Engler et al. 2003; Engler et al. 2012; Choo et al. 2014; Carter et al. 2012; Santillo et al. 2011; Johansson et al. 2007). These studies indicated that [^11^C]L-deprenyl-D_2_ can be used as in vivo marker for reactive astrocytosis, providing information concerning processes leading to neuronal loss.

To facilitate the studies of [^11^C]L-deprenyl-D_2_ in humans and small animals, we have developed an efficient synthesis of the precursor for [^11^C]L-deprenyl-D_2_ as well as its radiosynthesis. Previous work describes the preparation of the labelled precursor [^11^C]L-deprenyl-D_2_ from the activated d_2_-propargyl group and L-amphetamine as starting material. L-amphetamine is extremely hard to access to, especially because only few companies market it, as well as the import requirements given by the competent national authorities take long time and are difficult to succeed. Because of this, it is convenient to develop a new synthetic strategy.

In addition, the automated syntheses provide advantages over manual or semi-automated methods. Automated syntheses generally are more reproducible than manual and semi-remote syntheses minimizing the possibility of human errors. Therefore, an efficient alternative to the synthesis of L-nordeprenyl-D_2_ precursor of [^11^C]L-deprenyl-D_2_ as free base and an improved automated synthetic method have been developed. This paper describes both aspects of the improved synthesis of [^11^C]L-deprenyl-D_2_.

## Methodology and research design

### Organic synthesis

All chemicals and reagents were purchased from Aldrich, Merck and Dorwil. Analytical TLC were performed on silica gel 60F-254 plates and visualized with UV light (254 nm) and *p*-anisaldehyde in acidic ethanolic solution or iodine vapours. Column chromatography was performed using silica gel (SAI, 63–200 μm). NMR spectra were recorded on a Bruker DPX-400 spectrometer. The assignment of chemical shifts was based on standard NMR experiments (^1^H, ^1^H–COSY, HETCOR and ^13^C–NMR). The chemical shifts values were expressed in ppm relative to tetramethylsilane as internal standard. Mass spectra were determined on a Shimadzu DI-2010 (EI-MS) or Applied Biosystem API 2000 (ESI-MS). IR were obtained using a Shimadzu IR equipment Affinity-1 (Fourier Transform Infrared Spectrophotometer). Materials, instruments, protocols and documents used for precursor synthesis were in agreement with GMP recommendations.

### Synthetic procedures

(S)-2-Amino-3-phenyl-1-propanol (**1**):

i-a) A mixture of lithium borohydride (0.27 g, 12 mmol) in dry THF (6 mL) was cooled at 0 °C and trimethylsilyl chloride (3.1 mL, 48 mmol) was added subsequently. The ice/water bath was removed and the mixture stirred at room temperature for 20 min. Then, the mixture was again cooled to 0 °C and L-phenylalanine (1 g, 6 mmol) was added. The ice/water bath was removed, and the reaction mixture was stirred at room temperature for 12 h. The reaction mixture was cooled to 0 °C, and methanol (9 mL) was added dropwise, followed by aqueous sodium hydroxide (5 mL, 2.5 M). Finally, the mixture was evaporated *in vacuum*, and the residue extracted with chloroform (5 × 5 mL). The combined extracts were dried with Na_2_SO_4_, filtered, and evaporated *in vacuum*. The white solid obtained was dried under vacuum for 24 h to yield **1** (0.84 g, 92% yield).

i-b) To a solution of L-Phenylalanine methyl ester hydrochloride (300 mg, 1.39 mmol) in a 1:1 (*v*/v) mixture of water and ethanol (3.5 mL) was added slowly with stirring a solution of lithium borohydride (103 mg, 4.73 mmol) in the same solvent (3.5 mL) cooled externally in an ice/water bath. When the addition of borohydride was complete the mixture was stirred for 1 h at room temperature. Next, the solution was evaporated under reduced pressure and the residual aqueous solution treated first with sodium hydroxide and then with sodium chloride to saturate the solution before extraction with ethyl acetate (5 × 5 mL). The extract was washed with brine, dried over anhydrous Na_2_SO_4_, and evaporated under reduced pressure to yield **1** as white solid (0.172 g, 82% yield). ^1^H NMR (400 MHz, CDCl_3_) *δ* (ppm): 7.35–7.31 (m, 2H), 7.27–7.21 (m, 3H), 3.68 (dd, *J* = 4 Hz, *J* = 10.4 Hz 1H), 3.44 (dd, *J* = 7.2 Hz, *J* = 10.8 Hz, 1H), 3.18–3.12 (m, 1H), 2.84 (dd, *J* = 5.6 Hz, *J* = 13.6, 1H), 2.59 (dd, *J* = 8.8 Hz, *J* = 13.6 Hz, 1H), 2.02 (bs, 2H). IR (KBr): 3360, 3295, and 1580 cm^−1^; MS (ESI,) m/z: 152.1 (M^+.^ + H), 134.1 (M^+.^ - 18, H_2_O), 117.1 (PhCHCHCH_2_^.+^), 91.0 (PhCH_2_^.+^).

(S)-*tert*-Butyl (1-hydroxymethyl-2-phenylethyl)-carbamate (**2**): To a magnetically stirred suspension of **1** (1.0 g, 6.6 mmol) in water (6.5 mL) was added di-*tert*-butyl dicarbonate ((Boc)_2_O, 1.5 g, 9.9 mmol) at room temperature. After stirring for 25 min the reaction mixture, the white solid formed was filtered, washed with water and dried under vacuum for 48 h to yield **2** (1.31 g, 79% yield). ^1^H–NMR (CDCl_3_) δ (ppm): 7.35–7.31 (m, 2H), 7.27–7.23 (m, 3H), 4.76 (bs, 1H), 3.89 (bs, 1H), 3.72–3.67 (m, 1H), 3.60–3.55 (m, 1H), 2.87 (d, *J* = 7.2 Hz, 2H), 2.38 (bs, 1H), 1.44 (bs, 9H). IR (KBr) 3360, 1685, and 1525 cm^−1^; MS (ESI) m/z: 252 (M^+.^ + H), 235 (M^+.^ – OH, 17), 196 (M^+.^ - *tert-*butene, 56), 152 (M^+.^ – Boc, 101), 91 (PhCH_2_^.+^).

(S)-*tert*-Butyl (1-iodomethyl-2-phenylethyl)-carbamate (**3**): A mixture of iodine (1.59 g, 6.28 mmol), imidazole (0.47 g, 6.9 mmol) and triphenylphosphine (1.65 g, 6.26 mmol) in dry dichloromethane (50 mL) was cooled at 0 °C with stirring for 15 min. Next, the mixture was stirred at room temperature for another 15 min, and a solution of **2** (1.44 g, 5.71 mmol) in dry dichloromethane (18 mL) was added dropwise. The mixture was stirred for 15 min at room temperature; the solid formed was filtered and the organic layer washed with diluted aqueous Na_2_S_2_O_3_ and water, dried with Na_2_SO_4_ and evaporated in vacuo. After the workup, the crude was purified by column chromatography (SiO_2_, Hexane/EtOAc (9:1)), yielding derivative **3** as a white solid (1.6 g, 80%). ^1^H–NMR (CDCl_3_) δ (ppm): 7.35–7.32 (m, 2H), 7.29–7.25 (m, 3H), 4.72 (d, *J* = 7.2 Hz, 1H), 3.62 (bs, 1H), 3.44 (dd, *J* = 3.6 Hz, *J* = 10 Hz, 1H), 3.20 (dd, *J* = 4 Hz, *J* = 10 Hz, 1H), 2.96 (dd, *J* = 5.6 Hz, *J* = 13.2 Hz, 1H), 2.82 (dd, *J* = 8.4 Hz, *J* = 13.6 Hz, 1H), 1.46 (s, 9H). IR (KBr): 3350, 1690, 1525 cm^−1^. MS (ESI) m/z: 362.2 (M^+.^ + H) 306.1 (M^+.^ – *tert-*butene, 56), 105 (PhCHCH_3_), 91 (PhCH_2_^.+^), 57 (^+.^C(CH_3_)_3_).

(S)-*tert*-Butyl (1-methyl-2-phenylethyl)-carbamate (**4**):

A mixture of **3** (1.53 g, 4.24 mmol) in anhydrous tetrahydrofuran (32 mL) was cooled to −10 °C under nitrogen atmosphere. Next, a solution of sodium *tri-sec*-butylborohydride (N-Selectride) 1 M in tetrahydrofuran (6.36 mL, 6.36 mmol) was added dropwise and the resulting mixture was stirred at 0–5 °C for about 2 h. The reaction was quenched by the slow addition of water (3.0 mL) followed by the dropwise addition of a solution made by combining 45 mL of H_2_O, 3.0 g of K_2_CO_3_, and 23 mL of 10% H_2_O_2_. The reaction mixture was stirred at room temperature for 1 h. The THF was evaporated under reduced pressure, and the product was extracted with dichloromethane (4 × 15 mL). The organic layers were dried with Na_2_SO_4_ and the solvent evaporated in vacuo. After the workup the crude was purified by column chromatography (SiO2, Hexane/EtOAc (9:1)), yielding derivative **4** as a white solid (0.92 g, 93%). ^1^H–NMR (DMSO-*d6*) δ (ppm): 7.29–7.25 (m, 2H), 7.20–7.16 (m, 3H), 6.79 (d, *J* = 8.0 Hz, 1H), 3.68–3.61 (m, 1H), 2.75 (dd, *J* = 7.2 Hz, *J* = 13.2 Hz, 1H), 2.58 (dd, *J* = 7.2 Hz, *J* = 13.2 Hz, 1H), 1.34 (bs, 9H), 1.00 (d, *J* = 6.4, 3H). IR (KBr): 3360, 1687, 1520 cm^−1^. MS (ESI) m/z: 236 (M^+.^ + H), 180 (M^+.^ – *tert-*butene, 56), 119 (180 – NH_3_, OH, CO_2_), 91 (PhCH_2_^.+^).

(1,1-d_2_)-2-propyn-1-ol (**5**): A 1 M solution of LiAlD_4_ (29.0 mL, 29.0 mmol) in ether was cooled to −55 °C under nitrogen atmosphere in a two neck round-bottomed flask. Next, a solution of methyl propiolate (2.7 mL, 30 mmol) in anhydrous ether (10 mL) was added dropwise, over a period of about 60 min. The reaction mixture was stirred for another 90 min at −30 °C and was then allowed to warm to room temperature over a period of about 3 h and stirred overnight. Finally, the mixture was cooled to about 0 °C and quenched by the slow addition of water (1.5 mL) followed by the dropwise addition of a solution of NaOH (0.11 g in 0.75 mL) and 1 mL of H_2_O. The solid was allowed to settle and decanted. The solid formed was filtered, washed with ether (2 × 25 mL), the organic layers dried with Na_2_SO_4_ and the ether was evaporated under *vacuum*. d_2_-Propargyl alcohol was obtained as an oil (∼50% by 1H NMR signals) and was used in the next reaction without further purification. ^1^H–NMR (CDCl_3_) δ (ppm): 3.4 (s, 1H, OH), 2.4 (s, 1H, CH). ^13^C–NMR (CDCl_3_) δ (ppm): 60.4, 73.7, 81.0.

(1,1-d_2_)Propargyl *p*-toluenesulphonate (**6**): A mixture of **5** (crude mixture of the reduction process) and *p*-toluenesulfonyl chloride (5.8 g, 30 mmol) in anhydrous ether (70 mL) was cooled a − 10 °C under nitrogen atmosphere. Next, KOH (8.50 g, 152 mmol) was added and the mixture was allowed to warm to room temperature, over a period of about 1 h, and then stirred for 2 h. The solid decanted was filtered, washed with ether (20 mL) and the organic layer washed with brine, dried with Na_2_SO_4_ and evaporated in vacuo. After the workup the crude was purified by column chromatography (SiO_2_, Hexane/EtOAc (9:1)), yielding derivative **6** as a yellow oil (2.45 g, 40% two steps). ^1^H–NMR (CDCl_3_) δ (ppm): 7.85 (d, *J* = 8.4 Hz, 2H), 7.39 (d, *J* = 8.4 Hz, 2H), 2.49 (s, 1H), 2.48 (s, 3H). ^13^C–NMR (CDCl_3_) δ (ppm): 21.6, 57.1, 75.3, 77.3, 129.8, 130.1, 132.8, 145.1; MS (ESI) m/z: 235.1 (M^+.^ + Na).

**L-nordeprenyl-D**_**2**_: To a solution of **4** (117 mg; 0.50 mmol) in dichloromethane (1.0 mL) was added trifluoroacetic acid (0.25 mL) and stirred at room temperature for 2 h. The volatile components were removed under reduced pressure. Then, anhydrous DMF (5 mL), potassium carbonate (138 mg, 1.0 mmol) and d_2_-propargyl tosylate **6** (110 mg, 0.5 mmol) were added at room temperature. The resulting mixture was stirred at ambient temperature for about 24 h. The mixture was then diluted with water (20 mL) and extracted with diethyl ether (3 × 10 mL). The organic layers were combined, washed with brine, dried, and *concentrated* in vacuo. The resulting residue was then purified by flash column chromatography (hexane/ ethyl acetate (7:3)) to give the desired product (53 mg; 61%). ^1^H–NMR (CDCl_3_) δ (ppm): 7.35–7.30 (m, 2H), 7.26–7.22 (m, 3H), 3.24–3.16 (m, 1H), 2.76–2.64 (m, 2H), 2.19 (s, 1H), 1.65 (bs, 1H), 1.1 (d, *J* = 6.0 Hz, 3H). ^13^C–NMR (CDCl_3_) δ (ppm): 139.8, 129.3, 128.7, 126.2, 81.9, 71.1, 52.7, 43.0, 35.1, 19.5 MS (ESI) m/z: 198.2 (M^+.^ + Na), 176.2 (M^+.^ + H), 119.1 (PhCHCH_2_CH_3_^+.^), 91 (PhCH_2_^+.^), 58 (CHC-CD_2_-NH_3_^+.^).

### Radiosynthesis and quality control (QC) of [^11^C]L-deprenyl-D_2_

[^11^C]L-deprenyl-D_2_ was synthesized from [^11^C]MeOTf using a method previously described by our group [22]. Briefly, cyclotron produced [^11^C]CO_2_ is reduced to [^11^C]CH_4_, and further converted in [^11^C]MeOTf, using the commercial platform TRACERlab® FX C PRO (General Electric). [^11^C]MeOTf is transferred under helium stream to a small reactor where a solution of L-nordeprenyl-D_2_ (1.0 ± 0.2) mg in anhydrous MEK (Merck, 0.35 mL). Once the radioactivity in the reactor reached a plateau, solution was heated to 80 °C for 1 min. Crude [^11^C]L-deprenyl-D_2_ was separated from its precursor, the solvent and other minor radiochemical impurities using semipreparative reverse-phase HPLC (Nucleosil C18ec, 250 × 10, Macherey-Nagel; CH_3_COONH_4_ 0.1 M:MeCN 40:60, flow rate 6 mL/min, UV and gamma detection). The fraction containing the [^11^C]L-deprenyl-D_2_ was diluted in water (50 mL) for injection, passed through a SPE cartridge (Sep-pak C18 light), and eluted with EtOH (1 mL). [^11^C]L-deprenyl-D_2_ was formulated with saline (9 mL) and subjected to sterilizing filtration (0.22 μ).

Chemical and radiochemical impurities were detected and quantified using radio-HPLC: a mixture of TFA 0.1% and acetonitrile (75:25; *v*/v) was used as the mobile phase at a flow rate of 1.5 mL/min on a Nucleodur C18-ec 100–5 250 × 4.6 column (Macherey-Nagel). The whole HPLC analysis was completed within 10 min. The retention times of the L-nordeprenyl-D_2_ and L-deprenyl-D_2_ 4.4 ± 0.3 min and 5.4 ± 0.3 min, respectively. The chemical identity of [^11^C]L-deprenyl-D_2_ was determined by comparing the retention time of the unlabelled reference compound. The radiochemical purity was calculated considering the portion of [^11^C]L-deprenyl-D_2_ in relation to total radioactivity. The specific activity was determined considering total radiopharmaceutical activity and the amount of the unlabelled product.

The residual solvents (such as acetone, MEK and acetonitrile) and ethanol were analysed by gas chromatography (GC) in accordance with USP general chapter <467>. The appearance of the solution was checked by visual inspection, and pH was determined using a calibrated pH-meter. Radionuclidic purity was assessed by recording the corresponding gamma spectrum and radionuclidic identity by measuring the physical half-life.

Sterility and concentration of bacterial endotoxins were tested in accordance with USP general chapters <71>and <85>, respectively.

## Results and discussion

### Organic synthesis of L-nordeprenyl-D_2_

**Scheme 1:**
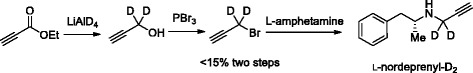
Conventional organic synthesis of L-nordeprenyl-D_2_ using L-amphetamine as starting material

**Scheme 2:**
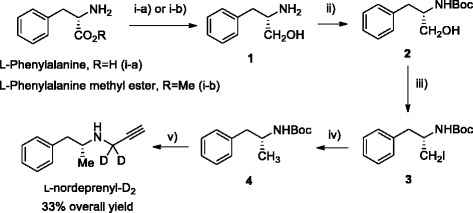
i-a) LiBH_4_, TMS-Cl, THF, r.t., 12 h, 92%; i-b) LiBH_4_, EtOH-H_2_O, r.t., 1 h, 82%; ii) (Boc)_2_O, H_2_O, 79%; iii) Triphenylphosphine, iodine, imidazole, CH_2_Cl_2_, 80%; iv) *N*-selectride, THF, 93%; v) a) TFA, CH_2_Cl_2_; b) **6**, K_2_CO_3_, DMF, 61% two steps

The synthesis of L-nordeprenyl-D_2_ was initially reported through direct *N*-alkylation reaction between L-amphetamine and propargyl bromide-α-α-D_2_ (Scheme 1) (MacGregor et al. 1988; Fowler et al. 1988). L-amphetamine was purchased commercially, while deuterated propargyl bromide was prepared by the reduction of methyl propiolate with LiAID_4_ followed by bromination with PBr_3_. Under this condition the deuterated key compound was obtained in low yield as a mixture difficult to purify, containing 15% of allyl bromide-1,1,3-D_3_. Another drawback of applying this methodology is the difficult access to L-amphetamine for use in research, as mentioned above. To avoid these problems, we have developed a new route to synthesizing L-nordeprenyl-D_2_ from L-phenylalanine in five steps (Scheme 2).

**Scheme 3:**
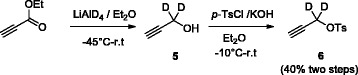
Organic synthesis of (1,1-d_2_)Propargyl *p*-toluenesulphonate **6**

Using this methodology, the key precursors to obtain are the derivative of L-amphetamine protected with Boc group, compound **4** (Scheme 2) and propargyl tosylate deuterated **6** (Scheme 3). First, to synthesize the derivative Boc-L-amphetamine **4**, a previously described synthetic sequence was adopted with some improvements in certain reaction steps (Quagliato et al. 2000; Gant and Sarshar 2010). At the beginning, L-phenylalanine as starting material was reduced in the presence of TMS-Cl and LiBH_4_, activating and reducing agent, respectively, yielding L-phenylalanilol **1** in excellent yield (92%, condition i-a, Scheme 2). In this context, when L-phenylalanine methyl ester was used as starting material and LiBH_4_ as a reducing agent, L-amino alcohol **1** was obtained in 82% yield and short reaction time (condition i-b, Scheme 2) (Hvidt et al. 1988). Subsequently the amino group of compound **1** was converted to *N*-*t*-Boc derivative by reaction with (Boc)_2_O in aqueous medium under mild conditions. The procedure was carried out using in short reaction times, and the L-Boc-phenylalanilol **2** was isolated by simple filtration in high yield (Scheme 2). Then, alcohol **2** was transformed into the iodomethyl **3** in presence of about 1 equivalent of triphenylphosphine-iodine-imidazole system under mild reaction conditions (15 min at room temperature). Subsequent reduction of iodomethyl derivative **3** using *N*-Selectride as reducing agent leads to the formation of L-Boc-amphetamine **4** in excellent yield. This last key intermediate was obtained with 54% overall yield following the synthetic methodology developed in this work. Considering that the next step requires the use of a propargyl deuterated derivative activated for *N*-alkylation reaction, we aimed obtaining the tosylate **6**, since this derivative could be easily isolated and purified by column chromatography. Thus, through a first reduction step of methyl propiolate with LiAlD_4_ the corresponding d2-propargyl alcohol **5** (Scheme 3) was obtained. The d2-propargyl tosylate **6** was efficiently obtained (40% in two reaction steps) from alcohol **5** by reaction with tosyl chloride in basic medium at room temperature. Finally, the precursor L-nordeprenyl-D_2_ was synthesized by a first step of deprotecting the derivative L-Boc-amphetamine **4** in the presence of TFA, followed by reaction of *N*-alkylation with d2-propargyl tosylate **6** using K_2_CO_3_ and DMF as solvent. The precursor as free base was stored in freezer at −20 °C, where its purity was 99.1% controlled by HPLC for 9 months (data not shown).

Through the development of this methodology it was possible to generate the L-nordeprenyl-D_2_ precursor with an overall yield of 33% in five synthetic steps and purity of 99,1% by HPLC and ^1^H–NMR analysis. The structure of the compounds synthesized was confirmed using analytical and spectroscopic techniques such as ^1^H NMR mono and bidimentional (COSY), ^13^C NMR and HETCOR (HSCQ and HMBC) experiments, IR and MS spectroscopy.

### Radiosynthesis of [^11^C]L-deprenyl-D_2_.

Radiosynthesis of [^11^C]L-deprenyl-D_2_ was initially reported using [^11^C]MeI as ^11^C–methylating agent (MacGregor et al. 1988; Fowler et al. 1988). Several radiosyntheses of ^11^C–labelled compounds have so far been improved by substituting [^11^C]MeI for [^11^C]MeOTf. In this context, Dolle et al. 2002; also reported a radiosynthetic procedure using [^11^C]MeOTf instead of [^11^C]MeI for [^11^C]L-deprenyl.

**Scheme 4:**
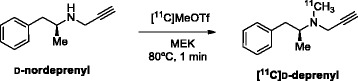
Radiosynthesis of [^11^C]D-deprenyl by one-step *N*-alkylation with [^11^C]MeOTf

We have recently described the fully automated synthesis of [^11^C]D-deprenyl tracer by one-step *N*-alkylation with [^11^C]MeOTf using the commercially platform GE TRACERlab® FX C Pro (Scheme 4) (Buccino et al. 2016). This methodology initially provided us great potential of [^11^C]MeOTf for reducing the amount of precursor and synthesis time, as well as for increasing radiochemical yields and reproducibility.

**Fig. 1:**
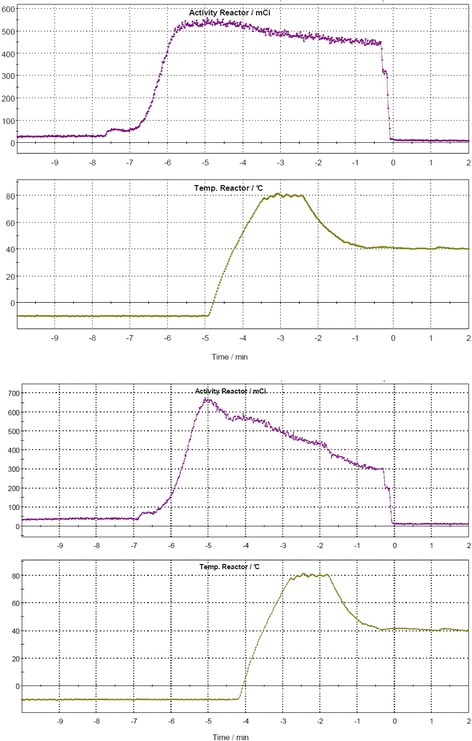
Radioactivity trapped in the reactor during the labelling step. Above: using L-nordeprenyl-D_2_ as free base. Below: using hydrochloride salt version of the same precursor

**Fig. 2:**
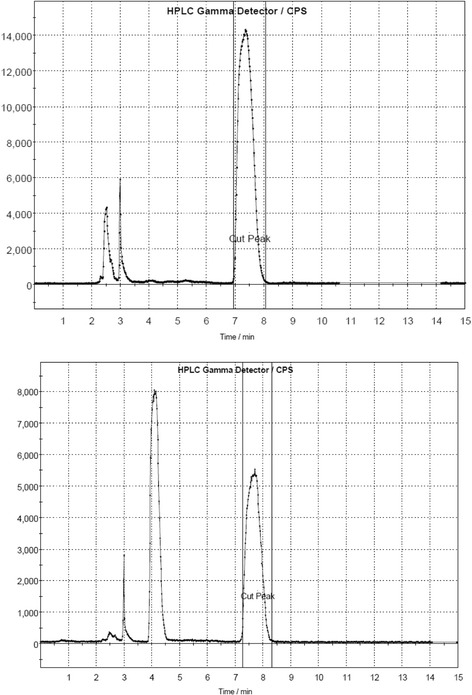
(above): semipreparative gamma chromatogram obtained with L-nordeprenyl-D_2_ free base and (below): same as above but using L-nordeprenyl-D_2_ hydrochloride salt. Peak in t_R_ = 7.5 min corresponds to [^11^C]L-deprenyl-D_2_

**Fig. 3:**
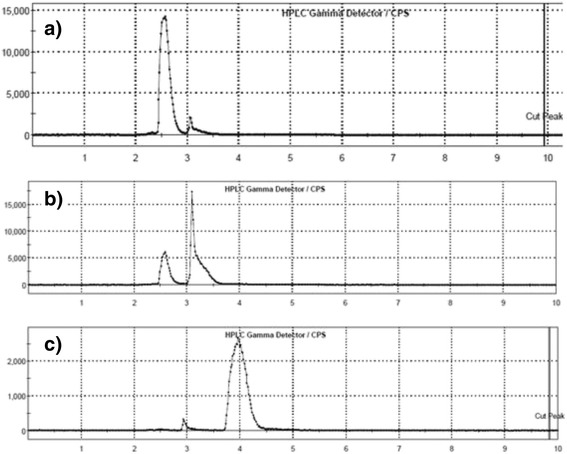
**a** [^11^C]MeOTf in MEK; (**b**) [^11^C]MeOTf in MEK/NaOH 3 M; (**c**) [^11^C]MeOTf in MEK/Benzalkonium Chloride 80%

The use of the free base version of the precursor D-nordeprenyl had a positive impact in the radiochemical yield of [^11^C]D-deprenyl. Because of these results, in the present work we proposed the use of the precursor L-nordeprenyl-D_2_ as free base for its labelling with [^11^C]MeOTf. Using the commercially available hydrochloride salt of L-nordeprenyl-D_2_, (Buccino et al. 2016), the overall radiochemical yield was 24 ± 9% (*n* = 10) (end of synthesis, decay corrected from [^11^C]MeI), but it increased to 44 ± 13% (*n* = 12) with the employment of the L-nordeprenyl-D_2_ free base (yields are referred to [^11^C]MeI, even when [^11^C]MeOTf is the radioactive precursor in the labelling reaction; TRACERlab®FX C Pro allows to measure activities of [^11^C]MeI but not those of [^11^C]MeOTf). The use of the aqueous NaOH to neutralize the hydrochloride salt is no longer necessary, and losses of radioactivity in the form of [^11^C]MeOH (possible product of hydrolysis of the radioactive precursor [^11^C]MeOTf) are diminished. This fact can be appreciated in the radioactivity profile trapped in the reactor during the labelling step (Fig. 1). We could observe an increased amount of [^11^C]L-deprenyl-D_2_ (peak at t_R_ = 7.5 min) in the semipreparative gamma chromatograph when free base precursor was used, being this compound more than 80% of the injected radioactivity. When the salt is used, this value decreased to less than 50%, and one major ^11^C–containing impurity at t_R_ = 4.0 min was found (Fig. 2). In order to confirm the identity of these radiochemical impurities observed during the radiosynthesis of [^11^C]L-deprenyl-D_2_ using the different precursors, a series of blank experiments were performed (Fig. 3). When bubbling [^11^C]MeOTf in anhydrous MEK (350 uL), after heating to 80 °C for 1 min., a major compound (95%) eluted at t_R_ = 2.6 min, which was assigned to unreacted [^11^C]MeOTf, and a minor compound (5%) at t_R_ = 3.0 min. That could correspond to [^11^C]MeOH (hydrolysis product of [^11^C]MeOTf). This minor peak increased its proportion when [^11^C]MeOTf is collected in MEK spiked with 3 uL of NaOH 3 M, as expected for a medium where basic hydrolysis is favoured.

We hypothesized that the major impurity observed for precursor L-nordeprenyl-D_2_.HCl might be [^11^C]MeCl, product of the nucleophilic attack of chloride anion to [^11^C]MeOTf. In order to confirm this assumption, [^11^C]MeOTf was collected in MEK containing 5 uL of 80% Benzalconium chloride (organic-soluble chloride salt). After heating to 80 °C for one minute, the chromatogram showed a major peak at t_R_ = 4.1 min, which validated our original hypothesis. Volatilisation of [^11^C]MeCl (Boiling point −23,8 °C, at 1 atm) during heating could explain the loss of radioactivity observed in this step when the precursor is in its hydrochloride form.

Radiochemical purity of [^11^C]L-deprenyl-D_2_ obtained using this methodology was 99.7 ± 0.6% (*n* = 12) and Specific activity was 170 ± 116 GBq/μmol (*n* = 12). Other QC parameters (such as ethanol and residual solvents concentrations, pH, half-life and radionuclidic purity) were in agreement with United States or European Pharmacopeas for all the batches produced with this methodology (*n* = 12).

These results are in concordance with those presented by Wilson et al. 2000; in which radiochemical yields of [^11^C]raclopride (from [^11^C]MeI) were very poor (<10%) when HBr salt of the radiolabelling precursor was used. These authors identified the major product as [^11^C]MeBr, which is less reactive than [^11^C]MeI for nucleophilic attack. Langer et al. 1999; also reported a similar finding when desmethyl-raclopride.HBr salt was used. In that case, when [^11^C]MeOTf is used as ^11^C–methylating agent, HBr salt of the precursor of [^11^C]raclopride only yielded [^11^C]MeBr as labelled product.

These findings allow us to conclude that the use of the free base form of the precursor of [^11^C]L-deprenyl-D_2_ presents many advantages in comparison to the hydrochloride salt, fundamentally in terms of radiochemical yield. Losses of radioactivity are decreased and radiochemical purity of crude [^11^C]L-deprenyl-D_2_ is increased, which affect dramatically the overall yield of the radiopharmaceutical process.

## Conclusions

A facile and efficient synthesis of L-nordeprenyl-D_2_ precursor of [^11^C]L-deprenyl-D_2_ as free base has been developed in five synthetic steps with an overall yield of 33%. The precursor as free base has been stable for 9 months stored at low temperature (−20 °C). An efficient automated synthetic method for [^11^C]L-deprenyl-D_2_ has been performed using L-nordeprenyl-D_2_ free base and [^11^C]MeOTf as methylating agent. This methodology offers a short preparation time (about 35 min) and simplicity in operation for routine preclinical and clinical studies.

## Abbreviations

(Boc)_2_O: Di-*tert*-butyl dicarbonate
Boc: (*Tert*-butoxycarbonyl)
CNS: Central Nervous System
COSY: Correlation Spectroscopy
DMF: Dimethylformamide
EtOAc: Ethyl acetate
HETCOR: Heteronuclear COSY
HPLC: High Pressure Liquid Chromatography
IR: Infrared spectra
LiAlD_4_: Lithium aluminum deuteride
MAO: Monoamine Oxidase
MEK: Methyl ethyl ketone
MS: Mass spectra
NMR: Nuclear Magnetic Resonance
PBr_3_: Phosphorus tribromide
PET: Position Emission Tomography
TFA: Trifluoroacetic Acid
THF: Tetrahydrofuran
TLC: Thin Layer Chromatography
TMS-Cl: Trimethylsilyl chloride
UV: Ultraviolet
